# The efficacy and safety of continuous intravenous infusion of rh-endostatin combined with platinum-based doublet chemotherapy for advanced non-small-cell lung cancer

**DOI:** 10.1007/s10637-024-01439-x

**Published:** 2024-05-03

**Authors:** Xinyi Liu, Zihan Guo, Lin Su, Anli Zuo, Min Gao, Xiang Ji, Jiameng Lu, Shuran Yang, Yunxiu Jiang, Degan Lu

**Affiliations:** 1 Department of Respiratory, Shandong Provincial Qianfoshan Hospital, Shandong Institute of Respiratory Diseases, Shandong Institute of Anesthesia and Respiratory Critical Medicine, The First Affiliated Hospital of Shandong First Medical University, 250014 Jinan, Shandong P.R. China; 2 Department of Respiratory, Jinan Fourth People’s Hospital, 250000 Jinan, Shandong P.R. China; 3 School of Microelectronics, Shandong University, 250100 Jinan, Shandong China

**Keywords:** Chemotherapy, NSCLC, Rh-endostatin, Continuous intravenous infusion

## Abstract

**Background:**

Platinum-based doublet chemotherapy is commonly used in the treatment of non-small cell lung cancer (NSCLC). A growing body of evidence indicates that incorporating antiangiogenic agents into platinum-based chemotherapy may enhance the survival outcomes for NSCLC patients. However, the optimal administration protocol for intravenous recombinant human endostatin (rh-endostatin), an antiangiogenic agent, remains uncertain at present.

**Aim:**

This study aims to investigate the efficacy and safety of 5-d continuous intravenous infusion of rh-endostatin in combination with chemotherapy for patients with advanced NSCLC. The predictive biomarkers for this treatment regimen were further probed.

**Methods:**

This prospective, single-arm multicenter study enrolled a total of 48 patients with advanced NSCLC who were histologically or cytologically confirmed but had not received any prior treatment from January 2021 to December 2022. Prior to the chemotherapy, these patients received a continuous intravenous infusion of rh-endostatin (210 mg) over a period of 120 h, using an infusion pump. The chemotherapy regimen included a combination of platinum with either pemetrexed or paclitaxel, given in 21-day cycles. The primary endpoint of the study was median progression-free survival (mPFS), and the secondary endpoints included median overall survival (mOS), objective response rate (ORR), disease control rate (DCR), and assessment of adverse events (AEs).

**Results:**

The mPFS was 6.5 months (95% confidence interval (CI): 3.8–9.1 m) while the mOS was 12.3 months (95% CI: 7.6–18.5 m). The ORR and DCR was 52.1% and 75.0%, respectively. Leukopenia (52.1%), anemia (33.3%), and thrombocytopenia (20.8%) were the most common adverse effects and these toxicities were deemed acceptable and manageable. In addition, a correlation was noted between elevated serum carcinoembryonic antigen (CEA) levels and decreased PFS and OS.

**Conclusions:**

The incorporation of a 5-day continuous intravenous infusion of rh-endostatin into platinum-based doublet chemotherapy has demonstrated both safety and efficacy in the treatment of advanced NSCLC. Furthermore, the baseline serum levels of CEA may potentially function as a predictor for the efficacy of rh-endostatin when combined with chemotherapy in NSCLC patients.

**ClinicalTrials.gov:**

NCT05574998.

## Introduction

Lung cancer is the most frequent cause of cancer-related death worldwide [1]. Non-small-cell lung cancer (NSCLC) accounts for approximately 85% of lung cancers, and a significant proportion of patients are diagnosed at an advanced stage (stage IIIB/IIIC/IV) with unfavorable prognosis. The five-year survival rates range from 4 to 17%, depending on the stage and the histologic type [2]. Although the introduction of immune checkpoint inhibitors (ICIs) alone or in combination with chemotherapy has expanded treatment options for NSCLC patients [3], platinum-based doublet chemotherapy remains the primary first-line therapy for patients with advanced NSCLC lacking driver gene mutations [4–6]. Nevertheless, the overall benefit of platinum-based chemotherapy for advanced NSCLC is limited [7].

Angiogenesis is a complex process that plays an important role in tumor growth and metastasis. Antiangiogenic treatment promotes normalization of tumor blood vessels, enabling effective delivery of chemotherapy drugs to cancer cells [8]. Bevacizumab, the first FDA-approved angiogenesis inhibitor for advanced NSCLC, is included in the NCCN guidelines for platinum-based protocols in advanced NSCLC [9]. In a large phase III clinical trial (ECOG 4599), the combination of chemotherapy and bevacizumab demonstrated improved progression-free survival (PFS) compared to chemotherapy alone in patients with advanced NSCLC [10]. Similar results were observed in the phase III BEYOND trial involving Chinese patients [11]. However, the use of bevacizumab is limited to patients with non-squamous NSCLC and may be associated with adverse effects such as hypertension, renal toxicities and thrombosis [12, 13].

Recombinant human endostatin (Rh-endostatin), an anti-angiogenic drug developed in China, has received approval for the treatment of advanced NSCLC patients [14]. Several clinical trials have revealed that chemotherapy plus rh-endostatin can effectively treat advanced NSCLC. For example, a phase III clinical trial investigating the use of rh-endostatin in combination with chemotherapy demonstrated improved overall survival (OS), with a mOS of 13.75 months compared to 9.77 months for chemotherapy alone in NSCLC patients [15]. Another study conducted by Han et al. reported that NSCLC patients treated with platinum-based chemotherapy plus rh-endostatin achieved a higher objective response rate (ORR) with a favorable safety profile [16].

Traditionally, rh-endostatin is administered through intermittent intravenous infusion of 7.5 mg/m^2^ once a day for 14 days. Continuous intravenous infusion of rh-endostatin using an infusion pump has been implemented in the treatment of NSCLC in China, but consensus on the optimal infusion scheme has not been reached [17]. Pu et al. reported that the combination of rh-endostatin, chemotherapy, and camrelizumab, an anti-PD-1 antibody, demonstrated a favorable efficacy and safety profile in patients with advanced NSCLC [18]. Additionally, a study utilizing 7-day rh-endostatin regimen showed good safety in the treatment of liver metastasis from gastric cancer [19]. Qin et al. conducted a retrospective study on the continuous intravenous infusion of rh-endostatin for 5 days in combination with chemotherapy in patients with recurrent advanced NSCLC. The results demonstrated improved adherence to the 5-day continuous intravenous infusion of rh-endostatin, as well as favorable efficacy and safety [20]. Based on the theory of the vascular normalization window, the optimal timing for the use of rh-endostatin is suggested to be 4–6 days prior to chemotherapy. From this perspective, a 5-day dosing regimen of rh-endostatin seems to be feasible [21, 22]. However, there is a lack of prospective studies on the 5-day dosing regimen in combination with chemotherapy as a first-line treatment for advanced NSCLC. Therefore, the purpose of this prospective study is to evaluate the efficacy and safety of a 5-day continuous intravenous infusion of rh-endostatin plus chemotherapy in patients with advanced NSCLC.

## Materials and methods

### Patients

Between January 2021 and December 2022, a total of 48 treatment-naive patients with histologically or cytologically confirmed stage IV advanced NSCLC were enrolled in this study. The inclusion criteria were as follows: (1) patients aged between 18 and 75 years old; (2) Eastern Cooperative Oncology Group (ECOG) performance status score of 0 to 2; (3) expected survival of at least 3 months; (4) presence of at least one measurable disease based on the Solid Tumors Response assessment Criteria (RECIST version 1.1) [23]; (5) absence of EGFR/ALK/ROS-1 alterations; (6) no prior radiotherapy or chemotherapy; (7) sufficient blood system parameters, including hemoglobin ≥ 90 g/L, absolute neutrophil count (ANC) ≥ 1.5 × 10^9/L, and platelet count ≥ 90 × 10^9/L; (8) adequate liver and kidney function, with serum bilirubin ≤ 1.5 × upper limit of normal (ULN), alanine aminotransferase and aspartate transaminase ≤ 2.5 × ULN, alkaline phosphatase ≤ 5 × ULN, and serum creatinine 60 mL/min or higher, or creatinine clearance within the normal range. Exclusion criteria included: (1) presence of cardiopulmonary diseases such as congestive heart failure, treatment-resistant hypertension, significant arrhythmia, or recent myocardial infarction; (2) active and serious infections; (3) coagulation disorders or bleeding tendencies; (4) poor compliance. The study was reviewed and approved by the ethics committee of The First Affiliated Hospital of Shandong First Medical University, and all 48 patients provided informed consent and were able to actively participate in the treatment.

### Intervention measures

In this study, patients received a continuous intravenous infusion of rh-endostatin at a dose of 210 mg over a period of 120 consecutive hours using an infusion pump. Each treatment cycle lasted for 21 days (q21d). The chemotherapy regimens varied based on the histological subtype of the NSCLC.

For patients with adenocarcinoma, the chemotherapy regimen consisted of carboplatin with an area under the receiver operating characteristic curve (AUC) of 5–6 (or cisplatin at a dose of 75 mg/m^2^) on day 4, in combination with pemetrexed at a dose of 500 mg on day 4, given every 21 days for a total of 4 cycles.

For patients with squamous cell carcinoma, the chemotherapy regimen included carboplatin with an AUC of 5–6 (or cisplatin at a dose of 75 mg/m^2^) on day 4, along with taxol at a dose of 175 mg/m^2^ on day 4, given every 21 days for a total of 4 cycles.

### Baseline and follow-up assessment

Prior to initiating treatment, patients underwent a comprehensive set of physical, laboratory, and radiological examinations as per the study protocol. Laboratory tests included routine blood tests, coagulation function assessments, electrolyte levels, liver function tests, renal function evaluations, and the measurement of tumor markers such as neutrophil to lymphocyte ratio (NLR), carcinoembryonic antigen (CEA), neuron specific enolase (NSE) and lactate dehydrogenase (LDH). For radiological evaluation, it was performed at the beginning of treatment and every 6 weeks after the start of treatment until disease progression.

### Clinical efficacy and safety evaluation

The primary endpoint was mPFS. The secondary endpoints included mOS, ORR, disease control rate (DCR), and adverse events (AEs). The therapeutic efficacy of the treatment was evaluated based on the RECIST 1.1 criteria, which classified the treatment response as complete response (CR), partial response (PR), stable disease (SD), or progressive disease (PD). The ORR was determined by tallying the number of cases with CR and PR, and subsequently dividing this total by the overall count of evaluable patients. AEs were monitored and graded according to the National Cancer Institute Common Terminology Criteria for Adverse Events (CTCAE version 4.0).

### Statistical analysis

All data were analyzed using GraphPad Prism version 9.0. mPFS and mOS were estimated by the Kaplan-Meier method, which allowed for the calculation of survival probabilities and construction of corresponding 95% confidence intervals (95% CI). Survival curves were generated using the Kaplan-Meier method. “Survival” and “surveyors” R packages were used to determine the cut off values for NLR, CEA, NSE, and LDH.

## Results

### General information

**Fig. 1:**
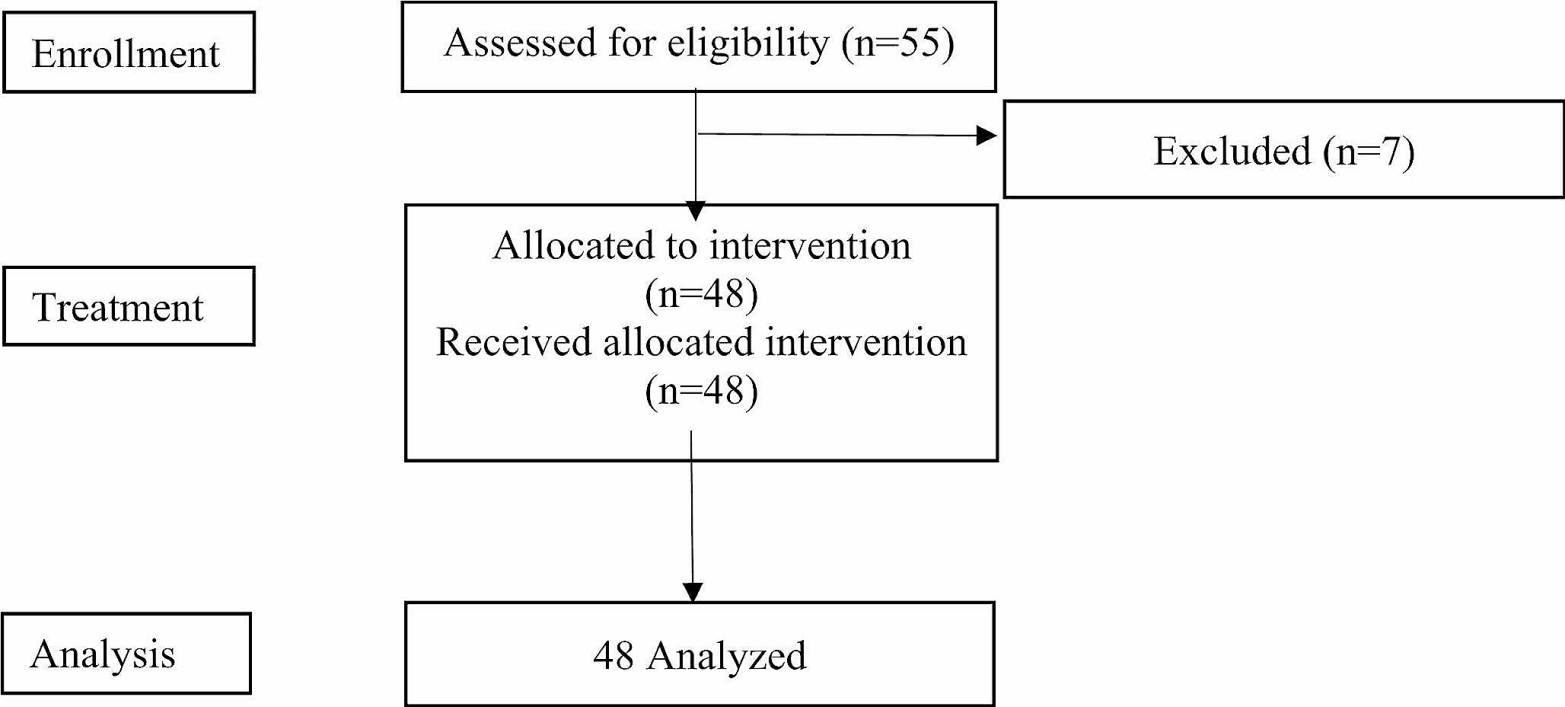
Consort diagram

A total of 55 patients with advanced NSCLC were screened and 48 patients were assigned to receive rh-endostatin plus chemotherapy between January 2021 and December 2022 in The First Affiliated Hospital of Shandong First Medical University. Seven patients were excluded from 55 screened patients for not meeting the inclusion criteria. As a result, a total of 48 patients were included in the final analysis (Fig. 1).

Table 1 presents the baseline characteristics of the patients. The median age of the patients was 66 years old, ranging from 47 to 75 years old. Among the participants, 30 were male and 18 were female. Additionally, 7 patients (14.6%) had squamous cell carcinoma, while 41 (85.4%) patients had adenocarcinoma.

### Clinical efficacy

**Fig. 2:**
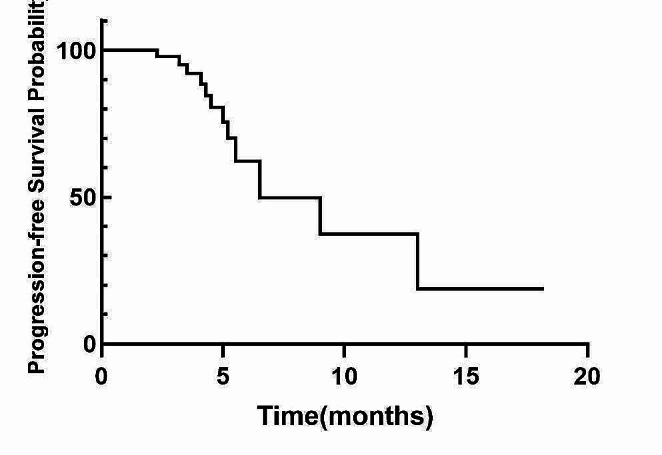
Kaplan-Meier curve of PFS

**Fig. 3:**
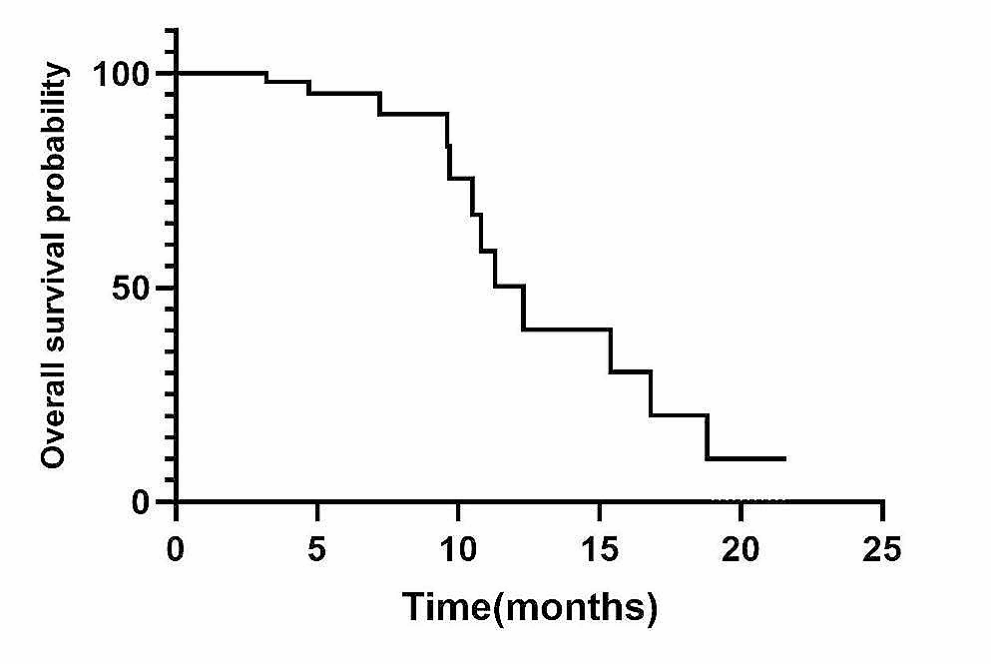
Kaplan-Meier curve of OS

Out of the 48 patients, 25 showed a PR, 11 demonstrated SD, and 12 experienced PD (Table 2). The ORR was 52.1% (25/48) and the DCR was 75.0% (36/48). There was no significant difference in the ORR and DCR between the patients who received taxol combined with platinum and those who received pemetrexed combined with platinum (*P* > 0.05). The study’s final follow-up was conducted on January 31, 2023. As shown in Figs. 2 and 3, the mPFS was 6.5 months (95% CI: 3.8–9.1 m) and the mOS was 12.3 months (95% CI: 7.6–18.5 m).

### Toxic effects

All 48 patients were included in the toxicity analysis. Table 3 lists the incidence of each adverse event. The majority of treatment-related adverse events were grade 1 or 2, including leukopenia (52.1%), anemia (33.3%), thrombocytopenia (20.8%), hypertension (4.2%), nausea (6.3%), vomiting (4.2%), and infection (2.1%). Major grade 3–4 adverse events were leukopenia (6.3%), anemia (2.1%), and thrombocytopenia (2.1%). Overall, the toxicities associated with rh-endostatin combined with chemotherapy in this study were deemed acceptable and manageable.

**Table 1:** The clinical characteristics of patients (*n* = 48)

Characteristics		Number(%)
Age, years median(range) Sex		66[47–75]
Female		18(37.5)
Male		30(62.5)
ECOG status		
0–1		43(89.6)
2		5(10.4)
Smoking Status		
Smoker		33(68.8)
Never smoker		15(31.2)
Pathological type		
Adenocarcinoma		41(85.4)
Squamous carcinoma		7(14.6)

**Table 2:** Therapeutic effect evaluation of NSCLC patients

Therapeutic effect	Patients (%)
PR	25(52.1)
SD	11(22.9)
PD	12(25.0)

PR, partial response; SD, stable disease; PD, progression disease.

**Table 3:** Occurrence of adverse reactions in NSCLC patients

Adverse reactions	All grades (%)	Grade 3–4(%)
Hematological toxicity		
Leukopenia	25(52.1)	3(6.3)
Anemia	16(33.3)	1(2.1)
Thrombocytopenia	10(20.8)	1(2.1)
Nonhematological toxicity		
Hypertension	2(4.2)	0
Nausea	3(6.3)	0
Vomiting	2(4.2)	0
Infection	1(2.1)	0

### Biomarker analysis

Further investigation was conducted to explore the relationship between serum biomarker levels and patient prognosis when combining rh-endostatin with chemotherapy for NSCLC. The results uncovered a significant correlation between elevated serum CEA levels and shorter mPFS and mOS. However, no substantial correlation was observed between serum levels of NLR, NSE, LDH, and patient prognosis.

**Fig. 4:**
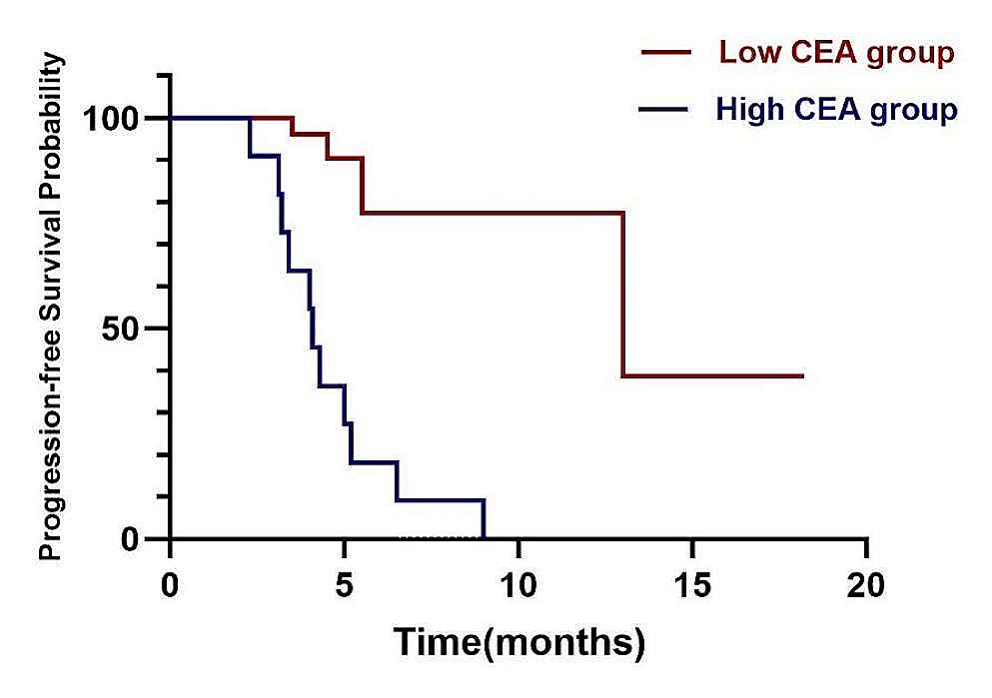
Kaplan-Meier curve of PFS for two groups

**Fig. 5:**
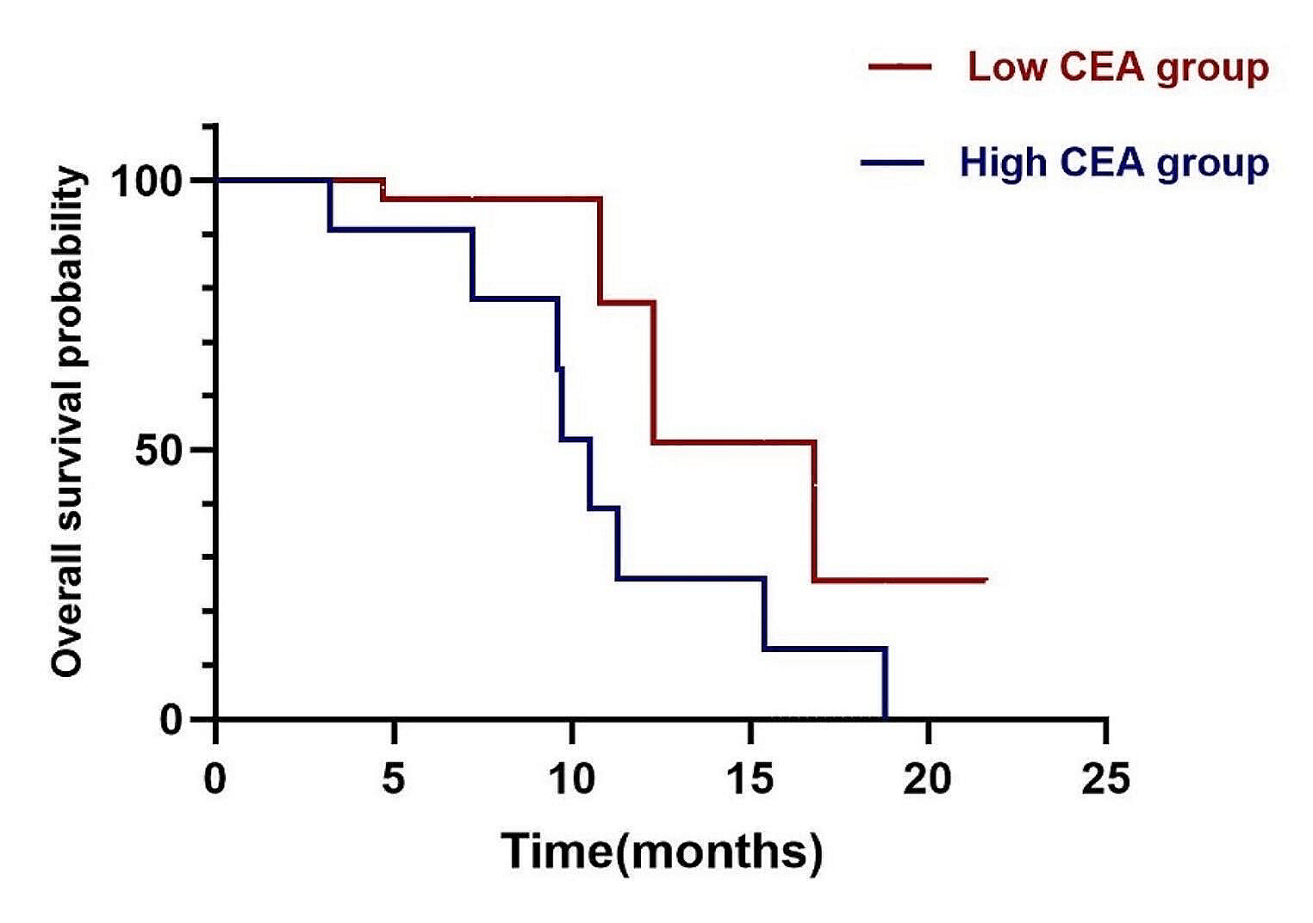
Kaplan-Meier curve of OS for two groups

During the analysis of patients’PFS and OS, a cut-off value of 5.5 ng/ml was established for serum CEA levels. Patients with CEA levels exceeding this cut-off were classified as the high CEA group, while the remaining patients were categorized as the low CEA group. The survival curves displayed a median PFS of 4.1 months for the high CEA group and 13.0 months for the low CEA group (*p* = 0.01, HR = 0.32, 95% CI: 0.10–0.99) (Fig. 4). Furthermore, the analysis of survival curves demonstrated that patients in the high CEA group had a significantly shorter median OS of 10.5 months in contrast to the 16.8 months observed in the low CEA group (*p* = 0.04, HR = 0.63, 95% CI: 0.19–2.08) (Fig. 5).

These findings suggest that initial serum CEA levels have the potential to serve as a promising biomarker for predicting the effectiveness of combining rh-endostatin with chemotherapy in NSCLC. Elevated serum CEA levels were correlated with shorter survival durations and unfavorable prognosis. This implies that evaluating serum CEA levels could be a valuable tool in guiding treatment strategies and forecasting prognosis for NSCLC patients undergoing this combined therapy.

## Discussion

Previous studies have provided evidence supporting the effectiveness of combining rh-endostatin with chemotherapy in improving PFS and OS in patients with advanced NSCLC [15, 20]. However, there is limited data available on the use of a 5-day intravenous infusion of rh-endostatin in combination with chemotherapy as a first-line treatment for advanced NSCLC. Therefore, this prospective study aimed to optimize the dosing regimen of rh-endostatin for previously untreated advanced NSCLC patients and evaluate the efficacy and safety of this treatment approach when combined with chemotherapy. The studys findings illustrated that the mPFS and mOS of NSCLC patients receiving this treatment regimen were enhanced. In addition, the toxicities were deemed acceptable and manageable. Overall, these findings add to the expanding body of evidence that supports the effectiveness and safety of combining a 5-day continuous intravenous infusion of rh-endostatin with chemotherapy as a viable first-line treatment for advanced NSCLC patients. To the best of our knowledge, this is the first prospective study investigating the use of a 5-day intravenous infusion of rh-endostatin in combination with chemotherapy as a first-line treatment for advanced driver gene-negative NSCLC.

In the current clinical practice, a combination therapy of anti-programmed cell death 1 (PD-1)/programmed cell death ligand 1 (PD-L1) antibodies and platinum-based chemotherapy has emerged as a widely employed first-line treatment for patients with advanced NSCLC that is unresectable and lacks driver mutations [24, 25]. In March 2019, the National Medical Products Administration (NMPA) of China approved the use of Opdivo and Keytruda in combination with chemotherapy as the first-line treatment for metastatic nonsquamous NSCLC in patients without EGFR and ALK mutations. However, due to the accessibility and expense of PD-1 antibodies, they were not included in our study. According to the IMpower150 study, the addition of atezolizumab to bevacizumab plus chemotherapy showed a significant improvement in PFS and OS among patients with metastatic nonsquamous NSCLC, regardless of their PD-L1 expression and EGFR or ALK genetic alteration status [26]. Therefore, our study offers an alternative treatment approach that combines PD1/PD-L1 inhibitor therapy for NSCLC patients without driver mutations, particularly for those with squamous NSCLC.

The results of this study demonstrated an ORR of 52.1% and a DCR of 75.0%. These findings are consistent with a study conducted by Sun et al., which examined the use of rh-endostatin combined with NP (vinorelbine + cisplatin) in NSCLC patients for the first time. They reported a response rate of 40% and a clinical benefit rate of 76.52% [15]. In a study by Cheng et al., the ORR and DCR for the continuous intravenous infusion of rh-endostatin in combination with first-line chemotherapy were 40.0% and 65.0%, respectively [27]. Furthermore, results from a large phase II clinical trial led by Han et al. revealed an ORR of 39.3% and a DCR of 90.2% in patients treated with rh-endostatin and paclitaxel-carboplatin (TC) [16]. In terms of prognosis and survival, Sun et al. reported a median time to progression of 6.3 months and a mOS of 13.75 months [15]. Han et al. reported that the mPFS and mOS were 7.1 versus 6.3 months (*p* = 0.522), and 17.6 versus 15.8 months (*p* = 0.696) in the treatment (TC + rh-endostatin) and control groups (TC + placebo), respectively [16]. Another study comparing different administration methods of rh-endostatin in NSCLC treatment demonstrated a mPFS of 6.0 months for patients receiving continuous intravenous infusion of rh-endostatin [27]. These results are in line with our findings in the present study.

Continuous intravenous infusion of rh-endostatin has been shown to extend the drug’s circulation time in the bloodstream and enhance its anti-tumor activity [28]. The use of portable infusion pumps for 120 h allows for continuous administration, ensuring that the anti-angiogenic treatment improves the accessibility of chemotherapy drugs to tumor cells [22]. In a study by Hansma et al., the safety of different doses of rh-endostatin was compared. It was found that a continuous intravenous infusion of rh-endostatin (210 mg) for 120 h was well-tolerated and related to the optimal time window for vascular normalization [22]. Furthermore, a study compared the efficacy and safety of continuous versus intermittent intravenous infusion of rh-endostatin in combination with chemotherapy for advanced NSCLC. The results revealed similar PFS and ORR between continuous and intermittent intravenous infusion of rh-endostatin (210 mg) [27]. Based on these findings, it can be concluded that a continuous intravenous infusion of rh-endostatin at a dose of 210 mg for 120 h is a feasible treatment regimen.

The combination of rh-endostatin and chemotherapy can result in common adverse reactions such as leukopenia, anemia, decreased appetite and hypertension [29]. In this study, grade 3–4 adverse reactions mainly consisted of leukopenia(6.3%), anemia(2.1%), and thrombocytopenia(2.1%) which were primarily related to chemotherapy. Similar grade 3–4 adverse reactions, including granulocytopenia (25.0%), anemia (5.0%), and thrombocytopenia (10.0%), were the most common in the study by Cheng et al. [27]. A meta-analysis indicated that angiogenesis inhibitors can potentially result in adverse reactions such as hypertension and myocardial ischemia [30], however the above adverse reactions were not observed in our study. Overall, the adverse events reported in the present study were manageable.

Rh-endostatin primarily targets the blood vessels of tumors rather than the tumor cells themselves. As a result, responsive patients may not experience immediate changes in tumor size following drug administration [31]. Identifying patients who will benefit from rh-endostatin is of utmost importance. Currently, there are no accurate predictors available to forecast the efficacy of rh-endostatin, although certain serum markers such as CEA and LDH have been proven to predict the efficacy of bevacizumab [32–34]. Gerald et al. confirmed that a high baseline CEA serum level predicted a poor outcome in patients with advanced colorectal cancer treated with bevacizumab [32]. In our study, we found that a low baseline CEA level was strongly associated with a longer PFS in NSCLC patients treated with rh-endostatin and chemotherapy. This finding is significant as it suggests that CEA levels could potentially serve as an outcome measure marker for rh-endostatin treatment in NSCLC patients. The possible reason behind this association is that overexpression of CEA enhances tumor angiogenesis. CEA induces pro-angiogenic behaviors in endothelial cells, including adhesion, spreading, proliferation, and migration in vitro, as well as tumor microvascularization in vivo independently of the vascular endothelial growth factor (VEGF)/VEGF receptor (VEGFR) system [35].

While this study provided valuable findings, it does have limitations. Firstly, the sample size was relatively small, although it was deemed sufficient to establish the conclusions of this study. However, larger sample sizes in future studies would provide more robust evidence. Secondly, this study was a single-arm study and did not include a control group for comparison. While comparing results with previous studies can offer some support for our conclusions, it is necessary to conduct controlled trials with larger sample sizes to validate our findings. Addressing these limitations through larger controlled trials would further strengthen the reliability and generalizability of the conclusions drawn from this study.

## Conclusions

In conclusion, our study findings indicate that the combination of a 5-day continuous intravenous infusion of rh-endostatin and chemotherapy is both effective and safe, providing a favorable treatment option for patients with advanced NSCLC. Furthermore, the baseline serum CEA levels show promise as a potential biomarker for predicting the efficacy of rh-endostatin combined with chemotherapy in this patient population. Our study provides an alternative choice for first-line treatment of driver gene-negative advanced NSCLC. Further research and validation in larger cohorts are necessary to firmly establish the clinical utility of CEA as a predictive biomarker for this treatment regimen.

## Data Availability

No datasets were generated or analysed during the current study.
